# Cytokine-Mediated Tissue Injury in Non-human Primate Models of Viral Infections

**DOI:** 10.3389/fimmu.2018.02862

**Published:** 2018-12-04

**Authors:** Cordelia Manickam, Spandan V. Shah, Olivier Lucar, Daniel R. Ram, R. Keith Reeves

**Affiliations:** ^1^Center for Virology and Vaccine Research, Beth Israel Deaconess Medical Center, Harvard Medical School, Boston, MA, United States; ^2^Ragon Institute of Massachusetts General Hospital, MIT and Harvard, Cambridge, MA, United States

**Keywords:** viral infections, cytokines, animal model, tissue damage, non-human primates

## Abstract

Viral infections trigger robust secretion of interferons and other antiviral cytokines by infected and bystander cells, which in turn can tune the immune response and may lead to viral clearance or immune suppression. However, aberrant or unrestricted cytokine responses can damage host tissues, leading to organ dysfunction, and even death. To understand the cytokine milieu and immune responses in infected host tissues, non-human primate (NHP) models have emerged as important tools. NHP have been used for decades to study human infections and have played significant roles in the development of vaccines, drug therapies and other immune treatment modalities, aided by an ability to control disease parameters, and unrestricted tissue access. In addition to the genetic and physiological similarities with humans, NHP have conserved immunologic properties with over 90% amino acid similarity for most cytokines. For example, human-like symptomology and acute respiratory syndrome is found in cynomolgus macaques infected with highly pathogenic avian influenza virus, antibody enhanced dengue disease is common in neotropical primates, and in NHP models of viral hepatitis cytokine-induced inflammation induces severe liver damage, fibrosis, and hepatocellular carcinoma recapitulates human disease. To regulate inflammation, anti-cytokine therapy studies in NHP are underway and will provide important insights for future human interventions. This review will provide a comprehensive outline of the cytokine-mediated exacerbation of disease and tissue damage in NHP models of viral infections and therapeutic strategies that can aid in prevention/treatment of the disease syndromes.

## Introduction

Microbial pathogens are constantly evolving to evade the host's immune system, and even with several decades of research and modern therapeutics, chronic diseases such as those caused by human immunodeficiency virus (HIV-1) and hepatitis C virus (HCV) are still globally prevalent. Viruses use multiple evasive strategies such as avoiding detection by pattern recognition receptors, T cell receptors and antibodies, mimicking or blocking cytokines, chemokines and other host proteins, and/or directly depleting immune cell subsets [reviewed in (1)]. Disruption of the cytokine milieu is also an important and commonly used strategy by viruses (2–4), since cytokines play important roles in shaping both innate and adaptive immunity. Cytokines are soluble proteins secreted by cells during inflammation that act as key mediators of immune cell recruitment and modulators of the immune response via a complex network of cellular interactions and signaling pathways. So far, more than 300 cytokines including chemokines, interferons (IFN), and lymphokines have been described (5). While cytokines can be broadly classified based on the nature of the immune response as pro-inflammatory cytokines such as interleukin (IL)-1, IL-6, type 1 IFN, tumor necrosis factor (TNF)-α, and anti-inflammatory cytokines such as IL-4, IL-10, and transforming growth factor (TGF)-β, they have pleiotropic functions whereby individual cytokines can have either pro- or anti-inflammatory properties according to the cell system involved.

In viral infections, cytokines play central roles in the development of protective anti-viral responses, but also potential immunopathology associated with chronic viral diseases. Viral interactions with host cellular receptors triggers pro-inflammatory cytokine secretion which are essential for viral clearance. However, dysregulations in the cytokine type and quantitative levels can lead to overactivation of immune cells, which in turn cause tissue damage leading to fatal complications. For instance, extensive characterization of IFN-α and its direct antiviral activity since its discovery in 1957 (6), has led to successful treatment of “non-A, non-B (NANB) hepatitis” even before the actual identification of HCV as the causative agent (7). Combination therapy of pegylated IFN-α with ribavirin was the standard therapeutic regime for chronic HCV-infected patients until the recent introduction of directly acting antivirals. However, IFN-α therapy can induce side effects such as fever and headache to severe life threatening conditions including thyroid, visual, auditory, renal and cardiac impairments, and pulmonary interstitial fibrosis (8). The therapeutic use of cytokines for infectious diseases, autoimmune diseases and malignancies, may also come at a steep price, since prolonged use of cytokines present severe side-effects due to the pleiotropic nature of these molecules (9–14). While, it is necessary to understand cytokine dysregulations in viral diseases to anticipate potential tissue injury and deterioration, their pleotropic, rapid, and in some cases local and long term tissue effects make the study of cytokines in humans challenging with potential development of fatal complications. These challenges can be met by the use of animal models. Animal models have been used since more than 2400 years and currently are employed in all areas of biomedical research including basic biology, infections, immunology, cancer, metabolic diseases, and behavioral studies (15). This review is primarily focused on the virus mediated cytokine dysfunctions in animal models specifically non-human primates (NHP), which are already fundamental in the validation of human data.

## Need for Animal Models in Studies of Viral Immunity

Much of what is known regarding antiviral immunity and tissue inflammation comes from studies conducted in animal models of human diseases. Animal models act as preclinical and translational gatekeepers since they allow the study of cellular interactions *in vivo* and elucidation of disease pathogenesis in tissues that may be difficult to access in humans. While mouse models have provided tremendous benefits to immunologists in understanding immune responses in humans, 65 million years of divergent evolution has contributed to significant differences in cytokines and cytokine receptors for the two species. Studies have shown poor correlation in genomic responses to acute inflammatory stress between humans and mice (16), and engagement of different chemokine/cytokine pathways in response to oxygen and glucose deprivation by human neurons compared to murine neurons (17). IL-13 seems to induce B cell class switching for IgE production specifically in humans whereas mice require IL-4 (18, 19). Similarly, IL-7 receptor deficiency inhibits development of all T and B lymphocytes in mice (20), but only T cells in humans (21). Furthermore, a number of pathogens like influenza, HIV, or dengue are highly tropic to their respective hosts and do not mimic human pathologies in mice, potentially restricting the use of mice as models for some infectious diseases [reviewed in (22)].

**Figure 1:**
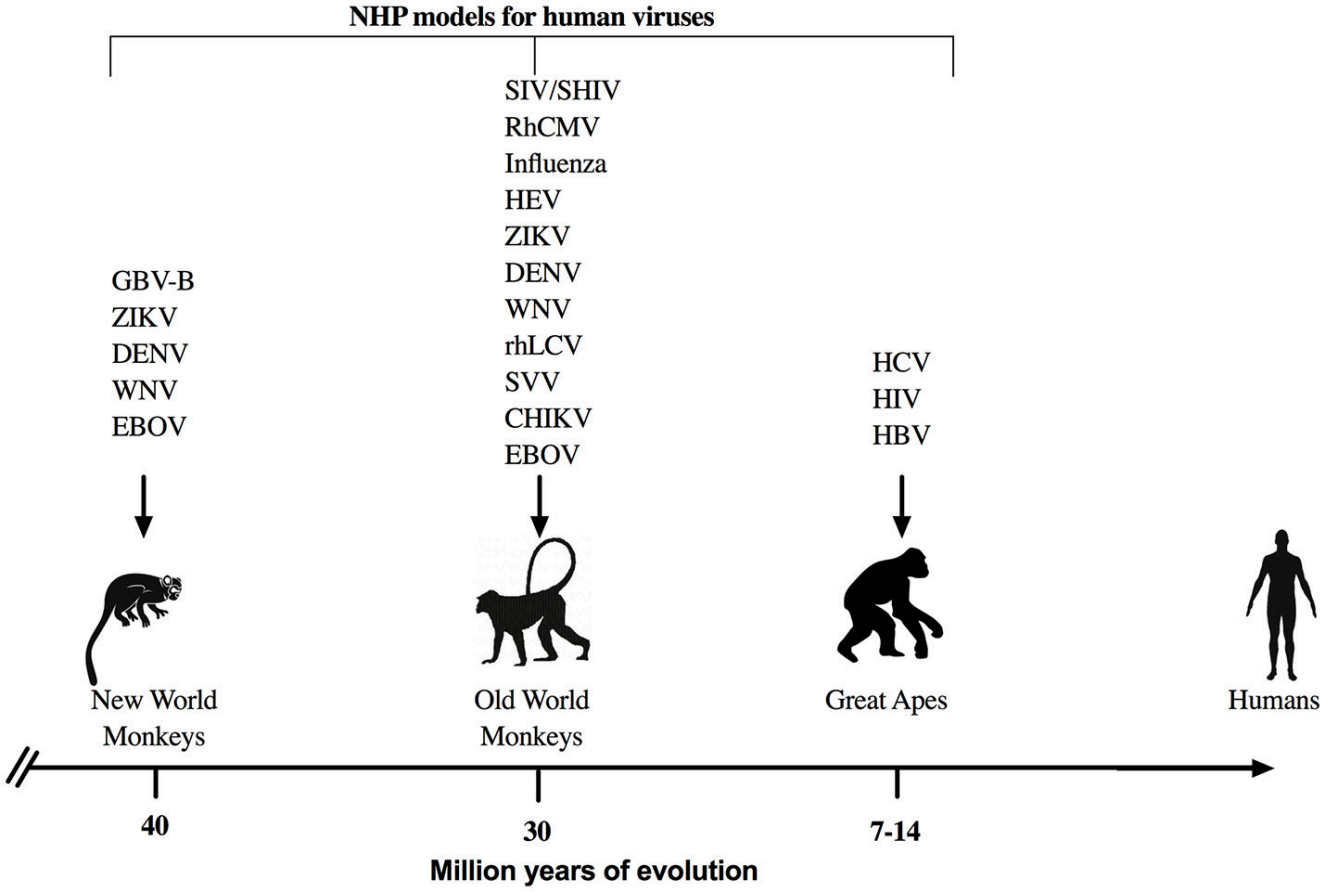
NHP models for viral infections. Representation of NHP models that are used commonly to study human viral infections with respect to the evolutionary divergence from humans. GBV-B, GB virus-B; ZIKV, Zika virus; DENV, Dengue virus; WNV, West Nile virus; EBOV, Ebola virus; SIV, Simian Immunodeficiency virus; SHIV, Simian/Human Immunodeficiency virus; RhCMV, rhesus cytomegalovirus; HEV, Hepatitis E virus; rhLCV, rhesus lymphocryptovirus; SVV, simian varicella virus; CHIK, chikungunya virus, HIV, Human Imunodeficiency virus; HCV, Hepatitis C virus; and HBV, Hepatitis B virus are some of the most common examples for viral studies in NHP.

NHP are perhaps the most commonly utilized models to study and understand immune responses against human infectious agents and for preclinical evaluation of therapeutics and vaccines (Figure 1). NHP have proven essential for research breakthroughs in maladies such as cancer, Parkinson's disease, heart diseases, and various infectious diseases such as HIV, Zika, Ebola, influenza, and others (23, 24). Even though NHP research accounts for < 1% of the all the biomedical laboratories working in animal models (24), the advantages offered by NHP due to the genetic and physiological homology to humans are manifold. Indeed, human and NHP cytokines are relatively conserved with 95% amino acid identity of most cytokines such as IL-2 and IFN-γ for Old World NHP and up to 90% amino acid identity for New World NHP (25). In addition, many cross reactive reagents and monoclonal antibodies for the detection of cytokines have been evaluated and validated for NHP species (NIH Non-human Primate Reagents Resource; http://www.nhpreagents.org) (25–28), making NHP attractive animal models to study viral pathogenesis and disease progression.

## NHP Models Commonly Used for Viral Diseases

### Great Apes

The great apes used previously as animal models include chimpanzees (*Pan troglodytes*), and to a lesser extent orangutans (*Pongo pygmaeus*) and gorillas (*Gorilla beringei*) (29). Chimpanzees share >98% DNA sequence homology to humans; and yet surprisingly, have immune systems that respond much more robustly to infections like HIV and hepatitis B virus (HBV). HBV and HCV can only pathogenically infect humans and chimpanzees, thus making chimpanzees, at one time, the primary animal model for therapeutics and vaccine research (30–32). However, the use of great apes in biomedical research has become increasingly restricted for ethical and cost reasons and therefore other NHP models are being increasingly utilized.

### Old World Monkeys

The Old World monkeys are primarily found in the continents of Africa, Asia, and Europe with rhesus macaques *(Macaca mulatta)*, cynomolgus macaques *(Macaca fascicularis)*, sooty mangabeys (*Cercocebus atys)*, African green monkeys *(Chlorocebus aethiops)*, and baboons *(Papio* spp.*)* being the predominant species used in biomedical research. Rhesus/cynomolgus macaques are perhaps the most widely utilized NHP animal models to study human infectious diseases. Besides HIV (33), macaque models have been used for infectious diseases such as influenza (34, 35), HBV (36, 37), HCV (38–40), measles (*Morbillivirus*) (41–43), cytomegalovirus (CMV) (44–46), among many others (47). Sooty mangabeys and African green monkeys are also used to study HIV and African green monkeys are used as a model for influenza (48). Less commonly used tree shrews *(Tupaia belangeri)* have also been explored as a model for HCV infection (49, 50).

### New World Monkeys

New World monkeys or neotropical primates include cotton-top tamarins *(Saguinus Oedipus)*, common marmosets *(Callithrix jacchus)*, owl monkeys *(Aotus lemurimus)*, and squirrel monkeys (*Saimiri boliviensis)*, which are commonly located in Central and South America. Although, the New World monkeys are more divergent than Old World NHP from humans, they provide a distinct advantage in biomedical research due to their relatively smaller size and lower cost compared to other NHP. Marmosets and tamarins have been used to study many flaviviruses such as HCV, Dengue, and Zika (51–56). Owl monkeys can be infected with Hepatitis E Virus (57) and at least some individual animals might have HIV-1 compatible CD4 alleles (58) making them potentially useful for HIV research. Squirrel monkeys have been utilized as animal models for HTLV-1 pathogenesis and vaccine development (59, 60) and as an experimental model for Nipah Virus (61).

## Cytokine Dysregulation in Viral Infection Models

### HIV/Acquired Immunodeficiency Syndrome (AIDS)

The emergence of HIV (Genus: Lentivirus, Family: Retroviridae) is the result of the combination of at least four simian immunodeficiency virus (SIV) transmission events from chimpanzees or gorillas to humans (62, 63). Therefore, SIV and simian/human immunodeficiency virus (SHIV) infections in NHP are commonly used to model HIV pathogenesis and development of vaccines and therapeutics. Specifically, rhesus macaques and sooty mangabeys have been critical in understanding the early phase of the infection (33, 64). Several studies (discussed below) have shown the principal involvement of an unusually vigorous immune activation leading to the progression and establishment of AIDS.

Based on plasma parameters from HIV-infected patients, the virus-mediated cytokine storm starts early in infection even before peak viremia is reached (65, 66). It rapidly initiates a cascade of events characterized by the production of the early pro-inflammatory cytokines, IL-15, and IFN-α, quickly followed by the more sustained TNF-α and monocyte chemoattractant protein (MCP)-1 during infection. Other pro-inflammatory cytokines like IL-6, IL-8, IL-18, and IFN-γ are elevated 2 days post the first wave of proinflammatory cytokines. At the same time, the secretion of IL-10, an immunoregulatory cytokine exponentially increases until it peaks at 5 days of infection (65). While, the IL-10/IL10-R pathway has a major role in preventing tissue damage observed during HIV infection by inhibiting Th1 responses and the production of anti-viral cytokines (IFN-α, IFN-γ, IL-2), it also contributes to viral persistence. Furthermore, the expression of the PD-1/PDL-1 pathway drives the inhibition of T cell function (67) and indirectly up-regulates expression of IL-10 (68). Indeed, blockade of PD-1 by anti-PD-1 antibody in infected rhesus macaques augmented SIV specific IFN-γ responses in CD8+ T cells in the blood, and could be synergized with vaccination and anti-retroviral therapies (69, 70). However, more NHP studies are necessary to establish the importance of PD-1 blockade particularly in mucosal tissues.

The magnitude of the cytokine storm is broadly associated with the clinical outcome in infected rhesus macaques and sooty mangabeys (66, 71). Indeed, the progressive infection in rhesus macaques is associated with production of IL-15, IL-18, IFN-γ, granulocyte-colony stimulating factor (G-CSF), MCP-1 and macrophage inflammatory protein (MIP)-1β but not in non-progressive sooty mangabeys (66). Similar cytokine dysregulation evidenced as elevated IL-12 has also been reported in HIV seroconverts (72) and South African women who are high risk population for acquisition of HIV infection (73). Furthermore, the cytokine storm leads to immune activation with global damage in mucosal tissues, specifically the gut and gut-associated lymphoid tissue (GALT) which are the early and major sites of virus replication (74). Specifically, the virus targets the IL-17/Th-17 pathway that is essential for preservation of the gut barrier, maintenance of the gut microbial environment, and prevention of translocation of microbial products into the circulation that could otherwise cause immune activation (75, 76). However, it is shown that cART can partially restore effective CD4+ T cells (more than 50% compared to non-treated) in the gut and enhance the Th17 subset which is associated with a better clinical outcome (77). This further illustrates the importance of NHP to study gut immunity in HIV infection and evaluate therapeutic modalities at mucosal tissues (78).

SIV infection in sooty mangabeys leads to a long non-progressive infection as observed in some HIV-infected individuals (79). Sooty mangabeys do not develop disease symptoms due to a low level of immune activation despite high level of viral replication (80). Instead of an inflammatory immune response, elevated regulatory T cells (Treg) and associated cytokines, TGF-β and IL-10 limit the level of immune activation (80). Similarly in infected African green monkeys, an anti-inflammatory environment is rapidly established due to increases in Treg frequency, TGF-β, and IL-10 levels in the plasma (81). Interestingly, a comparison of acute infection in African green monkeys and rhesus macaques revealed that a rapid and elevated IFN-α is triggered in both models but return to baseline levels after 28 days of infection was observed only in African green monkeys (82). Further, no changes in the levels of pro-inflammatory cytokines such as IL-6, IL-18, and TNF-α were reported in infected African green monkeys compared to uninfected controls (83). It was also shown that sooty mangabeys have a unique genome that protects them from developing AIDS (84). Of importance, these animals possess a different TLR-4 gene compared to NHP that develop AIDS. TLR-4 is a pattern recognition receptor that senses lipopolysaccharides on bacteria and initiates pro-inflammatory cytokine induction. HIV can induce microbial translocation that elicited exacerbated TLR-4 stimulation and lead to chronic immune activation (85, 86). Therefore the differential cytokine response and an overall lower immune activation, in part confers immune protection, less tissue damage and maintenance of gut barrier in non-pathogenic SIV infection of sooty mangabeys as well as African green monkeys (87, 88).

Rhesus macaques are not natural hosts of SIV infection and therefore, some SIV strains can induce strong viral load and the development of AIDS similar to HIV-infected individual (89). In a rhesus macaque cohort infected with pathogenic or non-pathogenic strains of SIV/SHIV, the progressor cohort exhibited low IFN-γ induced by CD4+ T cells compared to CD8+ T cells whereas, the non-progressor monkeys did not develop a similar immunomodulation (90). Furthermore, infection with virulent SIVmac251 strain directly upregulated the cytokine production (IFN-α/β, IL-12, IL-18) and led to the activation of natural killer (NK) cells which are one of the major antiviral innate immune cells and also act as a bridge to the adaptive system. Interestingly, the production of antiviral cytokines (IFN-α, IFN-γ, IL-2) was also associated with viral establishment (91). An over production of IL-7 in the gut during the early days of acute SIV infection in rhesus macaques could contribute to the cytokine storm by inducing elevated chemokine expression triggering immune cell recruitment (92). Overall, the cytokine storm induces a vicious cycle by spreading the infection and causing tissue damage due to an extensive inflammation in SIV progressive NHP models. To overcome this cytokine mediated disease exacerbation, several therapeutic formulations that use cytokines including IL-12, IL-15, and IL-2 or block cytokine receptors are increasingly being tested in SIV infection models (discussed in later section).

### Hepatitis B and C

Hepatitis B and C infections together are the leading causes of chronic liver disease worldwide (93). HBV (Genus: Orthohepadnavirus; Family: Hepadnaviridae) and HCV (Genus: Hepacivirus; Family: Flaviviridae) are hepatotropic viruses and cause both acute and chronic liver infections, which can progress to fibrosis and hepatocellular carcinoma. Interestingly, both viruses have a narrow host range (humans and chimpanzees) and have similar pathogenesis for progressive liver damage and persistence of infection. Studies in chimpanzees showed that HBV and HCV are not directly cytopathic (94–97) but instead cause liver injury due to chronic immune activation. Adaptive T cell and NK cell immunity are important in the control of viral hepatitis, but they can also prove detrimental in persistent infection. In cases of uncontrolled replication, infected hepatocytes secrete cytokines IL-8, CXCL-9, and CXCL-10, which recruit T cells to the infected liver, all correlating with histological damage (98–100). Further, innate immune NK cells are activated and recruited by high levels of IFN-α and IL-8 in the liver and induction of cytotoxic TRAIL pathway leads to killing of hepatocytes and liver injury (101). HCV-mediated liver inflammation is promoted by IL-1β and the TNF superfamily cytokines such as TNF-α, TNF-β, TWEAK, and LIGHT through the activation of NF-kB and MLCK-signaling pathways to reduce hepatocellular tight junction integrity (102, 103). In HBV infection, TNF-α secretion was associated with significant fibrosis, and IL-10 and IFN-γ were associated with necroinflammation (104). Additionally, as a result of viral overload, induction of interferon stimulated genes and elevated IL-8 and chemokines such as CCL2, CXCL1, and CXCL5 results in cholestatic HCV, which is associated with metabolic dysregulation (105, 106).

Due to the narrow host range, chimpanzees were critical for initially understanding the natural history and pathogenesis of HCV and HBV (32, 107). However, because of the limited use of chimpanzees currently, other surrogate animal models are being employed. To model HBV, cynomolgus macaques have been used but with an indirect infection approach: *ex-vivo* baculovirus-mediated HBV genome transfer in hepatocytes to cross the species barrier (108). Recently, a new virus called the capuchin monkey hepatitis B virus (CMHBV) has been discovered in Brazilian capuchin monkeys, a neotropical primate and has potential implications in the development of the much needed animals model for hepatitis B (109). The more commonly used NHP models for HCV are infections of neotropical primates, marmosets and tamarins, with the surrogate hepacivirus GBV-B of the same family Flaviviridae (51, 110, 111). Several studies showed that activated T cell immune responses and IFN-γ secretion are important for clearance of GBV-B (112, 113). However, similar to HCV-infected liver, immune activation correlated with liver damage in primary infections and re-infections in marmosets (114, 115). Activated NK cells expressing IFN-γ and perforin were accumulated in the liver and in addition elevated plasma IFN-γ and RANTES were associated with acute hepatitis in infected animals (114). Further, infected marmosets developed metabolic dysfunctions associated with GBV-B infection even after clearance of viremia indicating that viral hepatitis induces a cascade of events toward hepatic and systemic inflammation. Particularly, imbalance in levels of pro-inflammatory adipocytokines such as resistin and plasminogen activator inhibitor-1 secreted by dysfunctional adipose tissues contribute to local, systemic, and metabolic malfunctions (116). Given the importance of liver immune responses in progression of viral hepatitis, limited access to liver tissues has severely impeded development of HCV vaccine and HBV therapeutics.

### Zika

Infections with Zika virus (ZIKV; Genus: Flavivirus; Family: Flaviviridae) have recently caused a pandemic due to abortions, stillbirths, congenital birth defects, and neonate deaths called the congenital Zika syndrome (CZS) (117). ZIKV induced neuronal necrosis in the cortical layer of the brain is mediated by a complex array of cytokines and immune factors (118–120). While studies in brain tissue are limited, *in-situ* immunostaining of infected fetal brain samples showed that the predominant immune response was characterized by IL-4, IL-10, IL-33, iNOS, and arginase and therefore was generally skewed toward a Th2 response (118). IL-33, in particular is directly involved in pyroptosis, activation of inflammasomes, endoplasmic reticulum stress potentially leading to cellular damage (119). However, other cytokine responses indicative of Th1, Th17, Treg, Th9, and Th22 response were also involved to a lesser extent. Immune cells including microglia, CD4+ and CD8+ T cells, Treg, NK cells, M1/ M2 macrophages, and antigen-presenting cells contribute to the pathogenesis of the ZIKV induced inflammation (118). Thus, a complex relationship between different immune factors, cell damage, and direct viral action leads to ZIKV meningitis and encephalitis.

While ZIKV induced pathology and pathogenesis studies in humans are limited to samples obtained from autopsy of severe fatal cases, NHP have been tremendously helpful in elucidating pathogenesis and fast tracked development of several vaccine candidates (121–123). Indeed, fetal neuropathology, microcephaly, and other CZS symptoms were evidenced in several NHP models including rhesus, pigtail, and cynomolgus macaques, common marmosets, and squirrel monkeys infected during early pregnancy (55, 56, 124–128). Infection studies in common marmoset dams identified immune pathways in maternal viral responses. Interestingly, an increase in IFN-γ and pro-inflammatory cytokines as early as day 2 post-infection was reported. The pro-inflammatory response was maintained as elevated induction of type I/II IFN associated genes and pro-inflammatory cytokines even at day 7 post-infection and spontaneous abortion after 16–18 days of infection was reported with extensive viral infection in placenta and fetal tissues (56, 125). In infected rhesus macaques, viral persistence in the central nervous system and lymph nodes correlated with robust and early induction of pro-inflammatory responses and mTOR signaling pathways as evidenced by IFN-α induction at day 2, 4, and 6 post-infection and upregulation of transcript components of IFN-α and IFN-stimulated genes (ISGs) (OAS2, IFT1/2/3, ISG15, IRF7, IFI44, MX1, and MX2), pro-inflammatory cytokines and chemokines (TNF- α, IL-1, IL18, CCR7, CCL2, and CCL20), immunomodulatory pathways (IL-10, TGF-β, and T regulatory cells), and inflammasome pathways (NOD2, NLRP3, CXCL10, BTG2, BST2, OSM) at day 6 post-infection (129). As a result of these activated pathways, ZIKV persistence could contribute to the characteristic neuropathology associated with ZIKV. Further several experiments in NHP are currently underway for preclinical testing of vaccine candidates and Zika is an excellent example to illustrate the importance of NHP in developing vaccines within a short span of time.

### Dengue

Dengue virus (DENV; Genus: Flavivirus; Family: Flavivirdae), is a major vector borne disease in tropical and subtropical countries affecting approximately 100 million people worldwide, which can progress from the typical Dengue fever to fatal conditions such as Dengue hemorrhagic fever (DHF) and Dengue shock syndrome (DSS). Damage to vascular endothelium and uncontrolled activation of blood coagulation pathways in DHF can result in critical hypovolemic shock in DSS. Increased levels of cytokines, such as IFNs, IL-2, IL-8, TNF-α, and vascular endothelial growth factor A (VEGF-A) have all been reported to be associated with vascular leakage (130). Increased T cell activation and cytokine production in patients during both primary and secondary Dengue virus infections showed greater clinical severity of illness associated with cytokine storm characterized by elevated plasma pro-inflammatory cytokines such as IFN-γ, IL-6, IL-8, IL-10, CXCL9, CXCL10, CXCL11, MIF, TNF-α, and VEGF (130, 131).

Several NHP species are permissive to Dengue infection including chimpanzees, rhesus and cynomolgus macaques, sooty mangabeys, common marmosets, and owl monkeys, however the DENV induced hemorrhagic disease pattern is less common in NHP [reviewed in (132)]. In addition to elevated TGF-α and IFN-γ, increases in MCP-1, which drives immune cell recruitment, and potential cause of vascular damage was found elevated in rhesus macaques infected with DENV (133). A high dose intravenous inoculation of DENV induced classic dengue hemorrhage in infected rhesus macaques 3–5 days post-infection, with altered serum biochemical parameters indicative of coagulopathy (134). Similarly cytokine storm associated with enhanced dengue disease was detected in DENV infected marmosets, which showed a significant increase in plasma TNF-α as early as 3 days post-infection and significantly increased IFN-γ at 3, 6, and 20 days post-infection (52, 135). Indeed, antibody enhanced dengue disease in marmosets lead to CNS injury and was associated with intense TNF-α immunostaining in brain samples (135). Further, based on biomarker network analysis, two relevant strong axes during early stages of dengue fever were identified—a protective axis composed of TNF-α/lymphocytes/platelets, and a pathological axis IL-2/IL-6/monocyte/prothrombin time/viremia. Later time points post-infection showed the interaction of IFN-γ/platelets/DENV-3/prothrombin time, and the involvement of type-2 cytokines (IL-4, IL-5) (136). Overall, these studies indicate that elevated proinflammatory cytokines in dengue-infected NHP have a pathogenic role associated with disease severity.

### Influenza

Influenza A virus (Genus: Influenzavirus A; Family: Orthomyxoviridae) causes acute and severe respiratory illness in more than 1 billion people worldwide. The severity of influenza infection derives from the interplay between the virus and the host's ability to control viral infection and spread. In severe cases the host's response is hyperactivated and the resulting inflammatory response produces a cytokine storm (137–139) that is responsible for tissue injury and potentially death. This was seen during the 1918 H1N1 pandemic and more recently via the spread of H5N1. Endothelial cells from the lung have been implicated as key players in propagating the cytokine storm, in part from having elevated levels of CCL2, CCL5, and CXCL10 (140). Further inhibiting S1P1 receptor signaling on pulmonary endothelial cells, which leads to downregulation of cytokine/chemokine signaling, has been shown to decrease the development of cytokine storm following infection with influenza (140, 141).

One of the major issues in NHP modeling of influenza is the result of low animal mortality as compared to what happens in humans. While NHP can be infected with seasonal influenza strains they do not always display symptoms akin to those seen in humans (142). Influenza infection in NHP may lead to a biphasic subclinical fever early during the infection (143, 144), but this seems to be dependent on the mode of infection and dosage utilized (145, 146). Aerosol delivery using the full head chamber (145) results in a more lethal outcome, whereas the facemask leads to less severe symptoms. Infection with highly pathogenic influenza strains can induce clinical symptoms such as fever, cough and lethargy, and even showing signs of acute respiratory distress syndrome (124), bronchointerstitial pneumonia, peribronchiolar alveolitis, edema, and hemorrhaging (147–150). Further, in this model and others, increased levels of IP-10 (CXCL10), MCP-1 (CCL2), and IL-6 have been observed, which have been characterized as hallmarks of H5N1 human infection (138, 139, 151–153). Gene expression analyses have also shown that CXCL10 and CXCL11 are highly upregulated early during infection with highly pathogenic H1N1 and H5N1 and associated with elevated tissue damage (151, 152, 154). Using the full head chamber allows for the macaques to develop fulminant pneumonia that rapidly progressed to acute respiratory distress syndrome, which is the result of widespread alveolar epithelial cell death as well as depletion of alveolar macrophages.

### CMV

CMV (Genus: Cytomegalovirus; Family: Herpesviridae) can infect and persist lifelong in multiple cell types such as macrophages, neutrophils, fibroblasts, neuronal cells, hepatocytes and others (155–159). Human CMV (HCMV) infections are often reported in patients with suppressed immune system, including the elderly, AIDS patients, cancer patients, and transplant recipients. After infection, CMV hijacks cellular machinery, induces significant alterations in gene expression including IFN signaling genes, followed by a complex cascade of signaling events (160, 161) leading to upregulation of transcription factors like NF-κB and altered cytokine production, and thus successfully evades the host immune surveillance and disseminates to all organs (162–167). While the pathogenesis is not completely clear, elevated levels of MCP-1 and MIP-1α recruiting monocyte and macrophages to the site of infection could mediate tissue damage with uncontrolled viral replication in immunocompetent patients (168, 169). In congenital CMV infections, which cause severe birth defects in newborn babies, elevated MCP-1 and TNF-α in placenta could lead to adverse pregnancy outcomes or even death in utero (170, 171). Another group reported severe CNS abnormalities and brain vasculature damage in newborn babies due to proinflammatory cytokines IL-8, IL-6, TNF-α, and IL-1β upregulated by CMV infection of pericytes (172).

HCMV does not infect animals due to the species specificity of beta herpesviruses and interestingly the virus has co-evolved with its host species (173). Therefore, the study of specific CMV in their respective species of animal models has been helpful in elucidating CMV specific immunity. Indeed, simian CMV seroprevalence was reported in baboons, African green monkeys, and rhesus macaques as early as 1971 (174) and currently, rhesus CMV (RhCMV) infections in rhesus macaques is more commonly used as a NHP model (175). Since the global prevalence of HCMV ranges from 60 to 100%, animal models offer a unique advantage of being specific pathogen free, in this case CMV-free, in order to understand CMV immunity in comparison to uninfected population. RhCMV is particularly useful to model congenital infections (176) and co-infections such as CMV and HIV infections in the same host (177). Intrauterine inoculation of pregnant dams and intraamniotic/intracranial inoculations of the fetuses with RhCMV led to severe neurological defects and CNS lesion similar to HCMV (45, 176, 178). Further, RhCMV studies helped identify that the primate CMV encodes and expresses IL-10 homolog genes *in vivo* (179). Interestingly, the viral homolog had evolved functions that are beneficial to viral replication, primarily through immunosuppressive and anti-proliferative effects on host immune cells (179). The CMV IL-10 could also play a role in CMV's ability to subvert NK cell reactivity, thus avoiding NK cell lysis (179). Further, exploration of RhCMV infections in CMV free animals can identify immunopathogenesis pathways and therapeutic targets.

## Immunotherapeutic Approaches

Recombinant cytokines and anti-cytokine antibodies have recently gained traction in the pharmaceutical arena as a novel class of drugs for therapeutic purposes especially in autoimmune disorders and cancer (180, 181). There are few cytokine therapies that are already in use for therapy against viral infections such as IFN-α for HBV and HCV therapy. To overcome the severe side effects of IFN-α therapy, recently type III IFNs namely IFN-λ which have similar biological functions as IFN-α, have been tested preclinically in rhesus macaques (182). IFN-λ demonstrated antiviral effects similar to IFN-α without hematologic toxicity and thus could be used as an alternative therapy in chronic hepatitis patients. IL-12 administration has been previously studied in chimpanzees and rhesus macaques for understanding IL-12 mediated pathways and antiviral protection in SIV infections respectively (183, 184). IL-15 agonist, which has immunomodulatory functions, activates innate and adaptive immunity, and has been well characterized in NHP (185–188). Recently, a novel IL-15 superagonist ALT 803 potentiated T cell and NK cell responses leading to transient viral suppression in ART naïve SIV infected rhesus macaques (189). While the viral suppression was transient, this study illustrates IL-15 as a potential therapeutic agent particularly in combination therapy and ALT 803 is already in clinical trials for cancer therapy (190, 191). Even in DNA vaccine studies, IL-2 administration augmented vaccine elicited HIV-1, and SIV-1 specific immune responses in SHIV challenged rhesus macaques (192) thus showing that cytokine co-administrations can potentiate both vaccines and therapeutics.

Blocking of cytokine receptors or administration of cytokine antagonists can also be helpful in control of viral replication. Antagonists of CCR5 (maraviroc and vicriviroc) and CXCR4 inhibitor (Plerixafor) are relevant as they block HIV entry in cells and therefore can be used for HIV treatment (193). In addition to these small molecule CCR5 inhibitors, CCR5 blocking antibodies have also been characterized in preclinical rhesus macaques model of SIV infection (194–196). Further, maraviroc prevented cardiac dysfunction and cardiomyopathy associated with AIDS by blocking CCL5 and its recruitment of inflammatory macrophages in the heart tissue of SIV infected rhesus macaques (197).

Cytokine-based therapeutics are increasingly tested for other non-viral disease models of NHP. IL-13 neutralization for prevention of IgE mediated allergic responses in airway inflammation model of cynomolgus macaques (198), IL-6 receptor blocking and anti-TNF agent, infliximab for treatment of rheumatoid arthritis in cynomolgus macaques and rhesus macaques, IFN-α treatment effects in rhesus macaques model of cytokine induced depression (199, 200) are some of the few examples and could have potential applications in viral immunity and therapy. While cytokine therapy is advantageous in controlling viral replication or preventing tissue damage, systemic administration of cytokine, or cytokine blocking can result in altered hematopoiesis and immune activation, and severe complications due to the pleiotropic nature of cytokines in long-term therapy. Even in co-inhibitor receptors/checkpoint blockade therapy such as anti-PD-1 or CTLA-4 therapy commonly used for reversion of exhausted T cells in cancer and chronic diseases, undue immune activation or autoimmune responses is a primary risk leading to systemic or organ toxicities associated with uncontrolled inflammatory cytokine secretion and cytotoxicity by activated immune cells, which in turn require additional or follow-up immunosuppressive treatment [reviewed in (201, 202)]. Therefore, development of site directed biologics or cytokine therapy targeting viral infected tissues would be more beneficial than systemic administration.

## Conclusion

**Figure 2:**
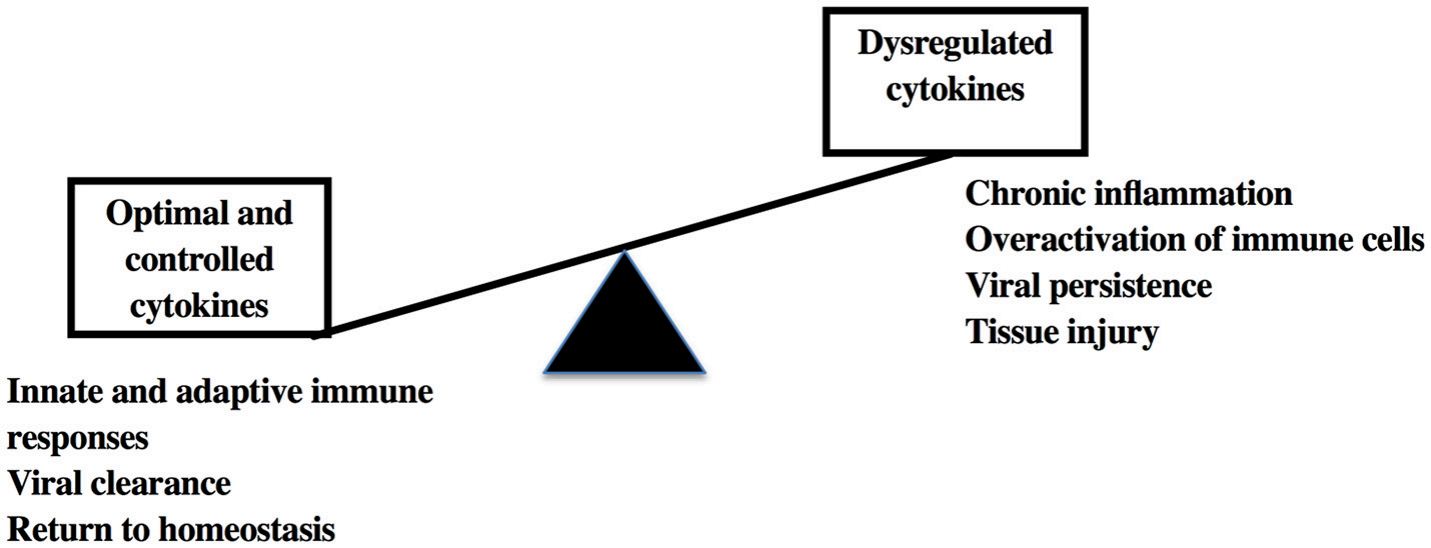
Cytokine responses and sequelae in viral infections.

Within the last few years, cytokines have been identified as key diagnostic, prognostic, and therapeutic agents in human diseases. Their multifaceted roles in immunity, tissue protection, and remodeling, maintenance of systemic and metabolic homeostasis make them important biomarkers for understanding and treating infectious diseases, cancer, auto-immune diseases, metabolic dysfunctions and other inflammatory processes. However, it is very important that their use in conjunction with other therapeutic and preventative strategies needs to be tested in pre-clinical models due to their propensity to cause immunopathology and tissue injury leading to serious complications in certain conditions (Figure 2). The usage of NHP models will be helpful for early prevention of tissue injury and associated autoimmune and metabolic syndromes that arise in diseases caused by viral and non-viral causes.

## Author Contributions

CM and SVS performed most of the writing. OL and DRR contributed to writing of specific sections. RKR oversaw overall preparation of the manuscript, contributed to writing, and edited the final version of the manuscript.

### Conflict of Interest Statement

The authors declare that the research was conducted in the absence of any commercial or financial relationships that could be construed as a potential conflict of interest.
